# Monolithic integration of Knudsen pumps to form a complete, self-sufficient fluidic system for microscale gas chromatography

**DOI:** 10.1038/s41378-025-01091-2

**Published:** 2025-12-09

**Authors:** Xiangyu Zhao, Tsenguun Byambadorj, Tao Qian, Qu Xu, Declan Winship, Yingkun Ma, Yutao Qin, Yogesh B. Gianchandani

**Affiliations:** 1 Department of Electrical Engineering and Computer Science, University of Michigan, Ann Arbor, MI USA; 2 Center for Wireless Integrated MicroSensing and Systems (WIMS²), University of Michigan, Ann Arbor, MI USA; 3 Department of Integrative Systems + Design, University of Michigan, Ann Arbor, MI USA

**Keywords:** Engineering, Chemistry

## Abstract

This paper reports the first microscale gas chromatography (μGC) system in which *all* fluidic components are monolithically integrated into a single 15 × 15 mm^2^ chip, including three Knudsen pumps, a preconcentrator, a separation column, and a capacitive detector. Knudsen pumps utilize thermal transpiration along narrow channels to induce gas flow, providing a compact, motionless solution that enables monolithic integration with high reliability and a long operational lifetime. The flow switching within the system is provided by a unique arrangement of multiple Knudsen pumps that eliminates the need for valves. To realize monolithic integration, the preconcentrator, separation column, and detector are arranged in a planar layout and are designed to be microfabricated using a common fabrication process. Methods to provide heat dissipation and thermal isolation are incorporated. In the experimental evaluation, the fabricated μGC system was operated to analyze the headspace of chemical mixtures, including alkenes, glycol ethers, aromatics, and mercaptans. The results showed a quantification accuracy of ±8.5% for the individual species within the mixtures, which is suitable for specific process monitoring applications in the chemical industry where concentrations of select target species and byproducts must be assessed continuously. The monolithic integration of gas pumps into a μGC system has not been previously reported and is an important step toward further miniaturization of μGC systems.

## Introduction

Chromatography has been used for process monitoring in the chemical industry since the 1960s^1^. Monitoring chemical reactions ensures catalyst health^2^, improves reaction safety^3^, and provides insight into catalytic mechanisms^4,5^. On-line process monitoring requires rapid, in situ, continuous, and quantitative results of a few known components in the reaction mixture^3,6^. Microscale gas chromatography (μGC) systems, with their compact size, low cost, low power consumption, and rapid response times^7,8^, meet the requirements for process monitoring.

A μGC system typically incorporates a preconcentrator for chemical collection and injection, a column for chemical separation, a detector for chemical detection, and pumps and valves to control gas flow^7^. Most μGC systems incorporate commercial pumps and valves^8–14^, whereas a few incorporate micropumps^15,16^ and microvalves^13^, or use customized architectures to avoid valves^15^. These components are commonly connected using the hybrid integration method, where the components are fabricated and assembled separately and then fluidically interconnected. However, hybrid integration increases system size and assembly costs, disadvantages mitigated by monolithic integration^17^. In the prior literature, the effort on monolithic integration of μGC systems has focused mainly on the analytical components, such as the integration of the column and detector^17–20^ or the preconcentrator, column, and detector^9,13,14^. However, there have been limited reports on the monolithic integration of valves and none on the monolithic integration of pumps. Consequently, μGC systems remain relatively high-cost, custom-built, partially miniaturized instruments rather than low-cost, mass-produced, and small chips.

The main challenges with monolithic integration of pumps and valves with other μGC components are the structural and fabrication incompatibilities. Most pumps and valves require flexible diaphragms and mechanical actuators^21–24^. These components are distinct from preconcentrators and columns, which are typically fluidic chambers or channels incorporating sorptive materials^18,25–28^. Different types of detectors may pose other requirements: suspended structures in thermal conductivity^18^ and nanocantilever detectors^29^, piezoelectric materials in surface acoustic wave sensors^30^, or coatings in chemiresistor arrays^11^ and capacitive detectors^15^. Additionally, monolithic integration introduces challenges in the thermal isolation between components because of their proximity and the high thermal conductivity in the commonly used silicon substrate^9^. Yield is another important factor that affects monolithic integration, as any nonfunctional component will render the monolithically integrated chip nonfunctional^9^.

Among different types of micropumps, the Knudsen pump is uniquely promising for monolithic integration due to its lack of moving parts. Operating on the principle of thermal transpiration, Knudsen pumps typically incorporate narrow pumping channels with a hydraulic diameter comparable to the mean free path of gas molecules. A temperature gradient along these narrow pumping channels transfers gas molecules from the colder end towards the hotter end^31^. Knudsen pumps have previously been incorporated into μGC systems by hybrid integration^15^, but never by monolithic integration. Some prior Knudsen pumps have used porous media^15,32^ to provide the narrow pumping channels, which are sandwiched between a Joule heater and a heat sink. Porous media Knudsen pumps are difficult to monolithically integrate because of the incompatibility between the porous media and standard microfabrication processes. Some other prior Knudsen pumps have incorporated lithographically microfabricated pumping channels with ultra-thin dielectric sidewalls, which are supported by a suspended dielectric membrane that also supports a heater^15,33–35^. For this type of pump, monolithic integration is also challenging due to the fragility of the suspended structures, which may be subject to yield and reliability issues in the subsequent fabrication, assembly, and testing steps. Additionally, the extra fabrication steps required to create the suspended ultra-thin sidewalls increase the fabrication complexity, because the fabrication of the other μGC components does not benefit from these steps.

This paper presents a μGC system that monolithically integrates three unidirectional Knudsen pumps with a preconcentrator, a separation column, and a detector into a 15 × 15 mm^2^ chip, achieving a monolithic gas sampling and analysis system (and hence named monoGSA). The Knudsen pumps in this work address the aforementioned manufacturability challenges by forming the pumping channels through a thick and unsuspended oxide layer, fabricated via standard deposition and etching processes. Although the unsuspended design provides a smaller temperature gradient for pumping, its consequence is mitigated by the co-design of other components in the monoGSA system, which incorporates narrower and shorter fluidic channels than other µGC systems. For applications unconstrained by power consumption, thermal crosstalk is mitigated by thermal isolation cutouts, heat dissipation from heat sinks and heat pipes, and forced convection from fans. The Knudsen pumps are coordinated to control gas flow magnitudes and directions required for chemical sampling and separation, eliminating the need for valves. The monolithic integration of micropumps with other μGC components into a single-chip system is a substantial advancement in the miniaturization of μGC systems.

## Design and fabrication

### System configuration

**Fig. 1:**
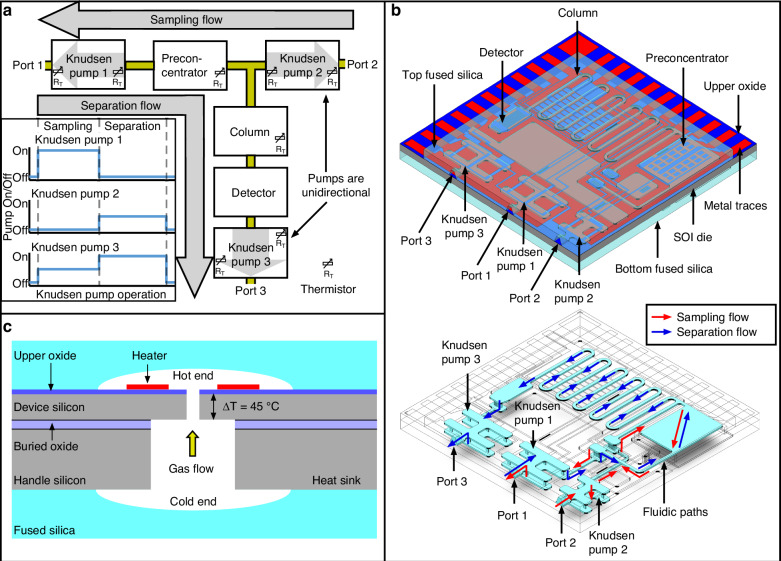
**Overview of the monoGSA system**. **a** The architecture of the monoGSA where three different Knudsen pumps operate together to control the gas flow of the system. **b** 3D view of the monoGSA chip showing the monolithic integration of preconcentrator, column, and detector with Knudsen pumps, along with a 3D view of the sampling and separation flow paths within the monoGSA chip. **c** Conceptual illustration of a KP pumping channel

Within the monoGSA chip, the sampling flow through the preconcentrator and the separation flow through the preconcentrator, column, and detector are controlled by the three unidirectional Knudsen pumps without the need for any valves (Fig. 1a, b). For sampling, Knudsen pump 1 (KP1) provides a pulling flow to draw chemicals from Port 2 into the preconcentrator, during which Knudsen pump 2 (KP2) is idle within the sampling path, whereas Knudsen pump 3 (KP3) provides a gentle counterbalancing pressure head that resists gas flow through the column and the detector. For separation, KP3 pulls the collected chemicals in the preconcentrator through the column and capacitive detector, while KP1 is idle, whereas KP2 provides a gentle counterbalancing pressure head that prevents additional gas from entering the system from Port 2.

The monoGSA chip is composed of a silicon on insulator (SOI) die sandwiched between two fused silica dies. The SOI die incorporates a 12 µm thick device silicon layer, a 0.4 µm thick buried oxide layer, and a 525 µm thick handle layer. The device silicon layer is covered by a 2 µm thick upper oxide layer, which is further covered by a Ti/Pt Metal 1 layer of 0.03/0.1 μm thickness and a Ti/Ni Metal 2 layer of 0.03/0.2 μm thickness. The SOI die incorporates pumping channels of the Knudsen pumps and through-holes for flow routing, all of which are formed through the silicon and oxide layers. Metal 1 provides all the on-chip heaters, thermistors, and detector electrodes, whereas Metal 2 provides the wire pads to form electrical connections and reduces the parasitic resistance for the metal layer connecting the wire pads to the heaters and thermistors.

The fused silica dies are 675 µm thick and incorporate 40 µm deep fluidic channels as well as 500 µm deep ports for capillary tube attachment. The top fused silica die forms fluidic channels for the separation column, preconcentrator, and detector. Both the top and bottom fused silica dies contain fluidic interconnect channels. Fused silica is selected as the material to form the µGC components and fluidic interconnect channels due to its lower metal content, which provides higher chemical inertness than glass. The locations of the μGC components are designed to minimize the length of fluidic connections, parasitic electrical resistances, and thermal crosstalk.

Knudsen pumps produce gas flow by thermal transpiration in narrow channels that have inner diameters smaller than or comparable to the mean free path. Along these channels, a temperature gradient drives the gas molecules from the cold side to the hot side (Fig. 1c). The three Knudsen pumps in the monoGSA are arranged to generate the gas flow selectively in the appropriate components of the system, while preventing unwanted gas flow in other components. As mentioned in Section “Introduction”, the manufacturing simplicity of each component is critical to the successful implementation of the overall monolithic system. Therefore, despite its higher pumping performance, we have chosen not to use our prior approach of building pumping channels with ultra-thin dielectric sidewalls supported by a suspended membrane^33–35^.

Instead, this work adopts a completely different Knudsen pump structure, which incorporates mechanically robust, easy-to-fabricate, unsuspended pumping channels. This Knudsen pump structure, while specifically designed for µGC integration in this work, has also been evaluated in its standalone form^36^. In this Knudsen pump structure, narrow pumping channels of the pump are formed by etching 1.2 × 200 μm^2^ openings through the upper oxide layer and the device silicon layer. Additionally, 30 × 230 µm^2^ openings are etched through the handle silicon to allow through-wafer gas flow. Metal traces closely surrounding the narrow channel in the upper oxide layer serve as Joule heaters to provide the hot side of the temperature gradient. With its lower thermal conductivity, the upper oxide layer accounts for most of the temperature difference and, therefore, the primary pumping effect. The device silicon and the handle silicon dissipate heat, forming the cool side of the temperature gradient. Using the upper oxide layer and device silicon to form the channel walls provides mechanical support for the pumping channel and improves the robustness and yield of the pump.

Multiple pumping channels are arranged in parallel to increase the output flow rate. They are placed as far as ≈1.5 mm apart to ensure sufficient heat sinking. Based on prior experimental evaluation of this Knudsen pump structure in its standalone forms, an input power of 0.5 W per channel is able to heat the hot side of the KP channel to 220 °C while the bulk silicon reaches 175 °C, creating a ΔT of 45 °C across the pumping channel. At an ambient temperature of 25 °C and ambient pressure of one atmosphere, this provides a blocking pressure of 570 Pa and the per channel maximum flow rate of 0.008 sccm (corresponding to a mass flow rate of 0.16 µg/s)^36^.

In the monoGSA system, KP1 provides the sampling flow, KP3 provides the separation flow, whereas KP2 provides a counterbalancing pressure to prevent flow through Port 2 during separation. Based on the gas flow needs, KP1 and KP3 each incorporate 6 parallel pumping channels with a total footprint of 3.8 × 2.1 mm^2^, whereas KP2 incorporates 4 parallel pumping channels with a total footprint of 2.3 × 2.1 mm^2^. The small dimensions of the pumping channels required for thermal transpiration make the pumps a significant source of flow resistance, which is partly mitigated by arranging the pumping channels in parallel. The details of the flow resistance of the monoGSA chip and the Knudsen pump performance are described in Section S1 of the Supporting Information.

The separation column is a serpentine flow channel with 200 μm width, 40 μm height, 4.5 cm length, and coated with 1 μm thick polydimethylsiloxane (PDMS) stationary phase. The separation column resolves individual gas species based on the differences in their interactions with the stationary phase. The relatively small column cross-section dimensions are selected for operational effectiveness at the modest flow rate from KP3. At a given flow rate, a smaller cross-section increases the flow velocity toward the optimal separation condition of the column. Although the smaller column cross-section causes a higher flow resistance and hence requires a higher pressure head from the Knudsen pump, this requirement is suited to the Knudsen pump design in this work. Additionally, the relatively small column height is designed to improve separation and compensate for the performance loss from the small column length that is limited by the chip footprint.

A capacitive detector is implemented in the monoGSA because of its advantages in simplicity and structural compatibility with the preconcentrator and column. The capacitive detector is a fluidic chamber that incorporates interdigitated electrodes coated with a sorptive polymer layer, similar to that in our prior work^15^. When gas enters the capacitive detector, it is absorbed by the polymer layer, and the resulting changes in polymer thickness and permittivity alter the capacitance, enabling gas detection. In this work, the interdigitated electrodes have 2 μm width and gap over a sensing area of 1.28 mm^2^ and are coated with 0.4 μm thick PDMS. This thickness of PDMS does not fully cover the fringe field between electrodes, allowing the swelling of the PDMS layer to serve as the dominant contributor to changes in capacitance. This is in contrast to thicker coatings where the swelling of the PDMS does not change the interaction with the fringe field, allowing the change in dielectric constant to serve as the dominant contributor to the response^15^. The electrodes are located on a 2 μm thick oxide layer for electrical isolation from the substrate silicon. The device silicon layer of the substrate is electrically grounded rather than floating; this grounding is provided via a pad of Ti silicide that forms a low resistance connection to the device silicon layer^37^.

Microfabricated preconcentrators typically incorporate packed sorbent particles^18,25,26^ or sorptive films^10,27,28^, which trap chemicals during sampling. For the subsequent analysis, the chemicals are thermally desorbed in a focused pulse and injected into the separation column. In the monoGSA, the preconcentrator is a 0.48 µL fluidic chamber with a heater and a thermistor. The heater is coated with a 4 µm thick PDMS, which serves as the sorptive film. As a material that has been previously demonstrated for preconcentration^27,28,38^, PDMS is selected in this work because the same material can also be used in the separation column and the detector, which facilitates the fabrication process. While the focusing provided by PDMS is a suitable compromise for this work, polymers such as Tenax TA may be suitable alternatives for enhanced preconcentration in other contexts^27,28^.

In this work, the preconcentrator is heated to 130 °C to desorb chemicals during separation. This temperature is significantly higher than the operating temperature of the other µGC components. Thus, it is essential to thermally isolate the preconcentrator to the extent that is possible within the constraints of the fabrication process and the available footprint of the die. A 200 μm wide grid of 30 × 230 µm^2^ thermal isolation cutouts with a separation of 30 μm is etched through the handle silicon surrounding the preconcentrator area to reduce the thermal conductance between the preconcentrator and the rest of the chip while preserving the mechanical strength of the monoGSA chip. Additionally, a heat dissipation package is designed, where an aluminum heat sink and a copper-water heat pipe are placed above and below the monoGSA, respectively. Thermal interface pads are used to provide a conformal contact between these elements. A fan provides a cooling flow for the heat sink.

Heat transfer simulations of the thermal isolation of the preconcentrator are provided in Section S2 of the Supporting Information. In the heat transfer simulations, the preconcentrator was heated rapidly to 130 °C for thermal desorption with a rise time of 2 s (Fig. S2b). The combined effect of the thermal isolation grid and the heat dissipation package was able to maintain the separation column temperature below 60 °C during desorption and reduce it below 40 °C within 5 s afterwards. Other μGC components exhibited similar temperature profiles. Overall, these thermal management methods allow individual components to reach sufficiently high temperatures for needed operation while sufficiently reducing the impact on the other integrated μGC components.

### Fabrication and assembly

**Fig. 2:**
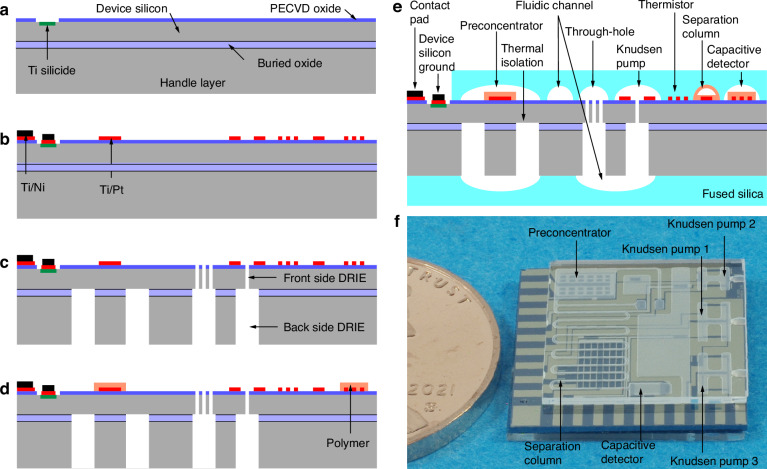
Fabrication flow of the monoGSA chip starting with a SOI wafer. **a** (Mask 1) PECVD and patterning of oxide, followed by (Mask 2) evaporation and annealing of Ti to form silicide. **b** (Mask 3) Evaporation of Ti/Pt metal and (Mask 4) Ti/Ni metal. **c** (Mask 5) Front side DRIE of device silicon layer and (Mask 6) back side DRIE of handle layer. **d** Polymer coating for the preconcentrator, column, and detector. **e** The cross-sectional view of the monoGSA chip after the top and bottom fused silica dies are attached to the SOI die. **f** Photograph of the microfabricated monoGSA chip

The monoGSA chip is lithographically microfabricated using a process involving eight masks, of which six are dedicated to the SOI die and two are dedicated to the fused silica dies. For the SOI die, the process starts with plasma-enhanced chemical vapor deposition (PECVD) of a 2 μm thick oxide layer on top of the device silicon (Fig. 2a). This oxide is patterned and wet etched to create an opening to the device silicon for silicide formation. Titanium is deposited by evaporation and patterned onto the exposed device silicon area by lift-off. The deposited titanium is then annealed using rapid thermal processing to 775 °C to form titanium silicide. The Ti/Pt and Ti/Ni metal layers are deposited by evaporation and patterned by lift-off using the third and fourth masks (Fig. 2b). A front-side deep reactive ion etching (DRIE) is performed using the fifth mask to form the through holes in the device silicon layer, and a back-side DRIE is then performed to etch through-holes in the handle layer (Fig. 2c).

The entire microfabrication process of the SOI die has been evaluated twice in two separate facilities. First, the process has been run in a university cleanroom (i.e., the Lurie Nanofabrication Facility at the University of Michigan). Next, the process has been repeated at a commercial foundry (WithMEMS, Gyeonggi-do, South Korea). Both attempts have been successful with high yields.

The two fused silica dies with 40 μm and 500 μm deep channels are micromachined by sandblasting using the seventh and eighth masks (performed by IKONICS® Corporation, MN, USA). The PDMS is coated on the SOI and fused silica dies in the targeted areas of the preconcentrator, separation column, and capacitive detector, followed by crosslinking (Fig. 2d). The fused silica dies are bonded to the SOI chip using a low outgassing epoxy (Epotek-377, Epoxy Technology Inc., MA, USA) (Fig. 2e). The final monoGSA chip has a footprint of 15 × 15 mm^2^ (Fig. 2f).

In preparation for testing, the monoGSA chip and its heat dissipation package are mounted onto a printed circuit board (PCB). Spring-loaded pins provide lead transfer between the chip and PCB. This PCB is then mounted on a motherboard that includes a microcontroller (#Raspberry Pi 3B + , Raspberry Pi Foundation, UK) as well as electronics for control and readout. The details of the electronics are provided in Section S3 of the Supporting Information. The overall system has dimensions of 14 × 12.5 × 7 cm^3^.

## Experimental results and discussion

### Evaluation of the monoGSA system

#### Test setup and operation control

**Fig. 3:**
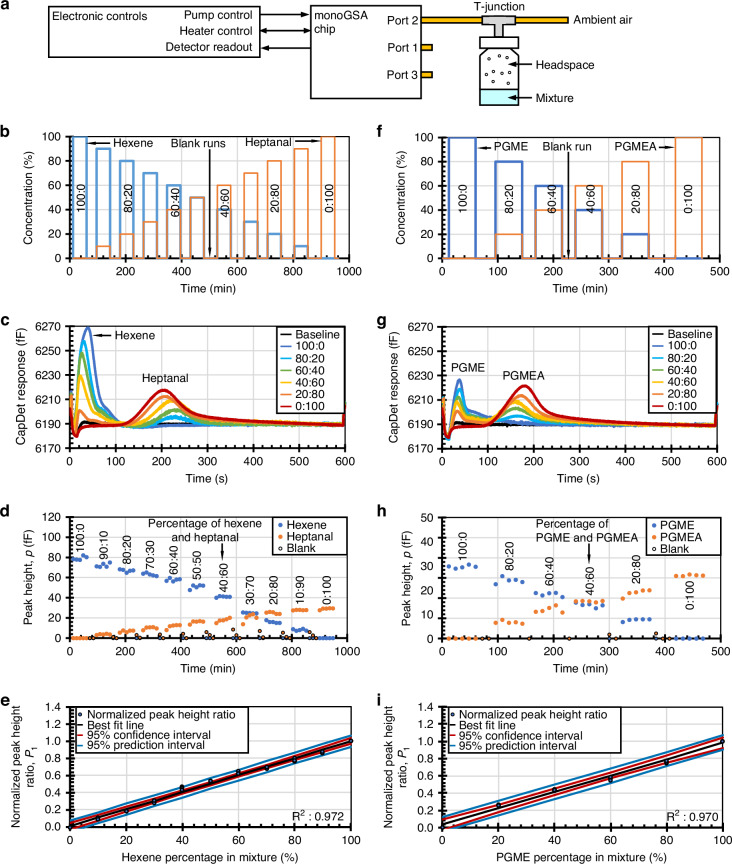
**Chemical tests with the monoGSA system. a** Schematic of the monoGSA test setup. For Mixture 1 (hexene and heptanal: The (**b**) concentrations tested, (**c**) chromatogram results, (**d**) peak heights from repeated tests, and (**e**) normalized peak height ratio for different concentrations. For Mixture 2 (PGME and PGMEA): The (**f**) concentrations tested, (**g**) chromatogram results, (**h**) peak heights with repeated tests, and (**i**) normalized peak height ratio for different concentrations

The test setup consists of the monoGSA chip, a sample vial, and electronic controls (Fig. 3a). Chemical mixtures were prepared and stored in a 2 ml sample vial. The headspace of the vial was connected to a metal T-junction, the other two ports of which were connected to Port 2 of the monoGSA and left open to the ambient, allowing the headspace of the vial to be sampled. The sample vial simulated the samples that can be extracted throughout a chemical reaction for in situ reaction monitoring^5^. The flow rate was monitored using a flow meter (MW-5SCCM-D/5 M, Alicat Scientific, Inc., AZ, USA), for which the measurement uncertainty was estimated to be ±0.001 sccm.

An analysis run consisted of two steps, sampling and separation, with the analysis conditions described unless specified otherwise. During sampling, KP1 was powered at 2.10 W to provide the sampling flow, and KP3 was powered by 0.84 W to resist parasitic flow through Port 3. The sampling flow rate was measured to be 0.011 sccm and the flow through Port 3 was 0 sccm. The sampling was performed for two minutes. With the heat sink, heat pipe, and fan all providing cooling, the chip temperature at the column and preconcentrator remained below 40 °C during sampling.

The separation step was performed after the sampling step for a duration of ten minutes. The preconcentrator was powered with 26.5 V to heat the preconcentrator area to 130 °C for 18 s, whereas the separation flow was turned on 2 s after the start of preconcentrator desorption. During separation, KP3 was powered by 2.10 W to provide the separation flow and KP2 was powered by 0.61 W to resist a flow through Port 2. The separation flow rate was measured at 0.009 sccm and the flow through Port 2 was minimized. Specifically, a gentle outward flow (fluctuating between 0.000 sccm and 0.001 sccm as indicated by the flow meter) was maintained to prevent unwanted inflow into the system. The column temperature rose to no more than 70 °C during the preconcentrator desorption and fell quickly to below 35 °C for the rest of the separation. With the cooling components, a 61 °C difference between the preconcentrator and column temperatures was observed during preconcentrator desorption. The measured temperature was 10.2% higher than the simulated temperatures for the preconcentrator during desorption but otherwise matched the simulated temperatures (Fig. S3).

#### Chemical tests

The monoGSA chip was designed to analyze reagents, products, or significant byproducts of known catalysis reactions to monitor reaction progress and catalyst health, such as esterification, hydroformylation, and chlorination. Esterification is widely used to produce esters that are incorporated in fragrances, flavors, solvents, and plastics^39^. As an equilibrium-limited reaction, esterification is constrained by the thermodynamic balance between reactants and products, necessitating process intensification or equilibrium-shifting strategies to increase conversion^40^. For these strategies, real-time in situ monitoring of reactant and product concentrations can be highly beneficial, as it provides necessary information for precise control over the reaction kinetics and hence ensures high yield and product purity. In addition to esterification, hydroformylation is used on a large scale, producing approximately 10 million metric tons per year of key intermediates for alcohols, esters, and other bulk chemicals^41^. Chlorination plays a role in the synthesis of over 200 FDA-approved pharmaceuticals^42^. Real-time, continuous monitoring of these reactions is also beneficial for ensuring safety, maximizing yields, and maintaining product quality.

Since multiple reactions are often monitored concurrently, arrays of GC systems are typically required. However, traditional benchtop gas chromatography systems are ill-suited for this task due to their large size, slow analysis times, and high cost, underscoring the need for a miniaturized, low-cost solution. The suitability of the monoGSA for process monitoring is demonstrated through its ability to quantify key reactants and products, operate across a wide volatility range, maintain accuracy under varying humidity, and consume minimal power.

Another potential application of the monoGSA is energy-efficient, extended monitoring. This can be achieved by reducing power consumption during sampling, either through passive diffusion or by operating the sampling pump KP1 at a fraction of its normal power. While the benefits of low-power sampling are minimal for short-duration experiments, they become significant over extended periods, where sampling can dominate total energy consumption. Energy-efficient operation is valuable for extended monitoring applications, like natural gas leak detection^43^ and other long-term environmental or industrial monitoring tasks. The suitability of monoGSA for extended monitoring applications is demonstrated through testing the sampling performance of a diluted sample over a long time period at passive or low sampling pump powers.

To test the system performance for these applications, a number of test mixtures were prepared. Mixture 1 contained hexene and heptanal to represent the reagent and product of the hydroformylation reaction^41^. Mixture 2 contained propylene glycol methyl ether (PGME) and propylene glycol monomethyl ether acetate (PMGEA) to represent the reagent and product of an esterification reaction^44^. Mixture 3 contained benzene, toluene, chlorobenzene, and 1,3-dichlorobenzene to represent a hydrodealkylation reaction followed by a chlorination reaction^45^. Additionally, Mixture 4, containing tert‑butyl mercaptan and tetrahydrothiophene, which are commonly used as odorant additives to natural gas, was prepared to investigate extended monitoring applications. The Kovats retention indices (which are unit-less metrics indicating the analyte retention times relative to n-alkanes) and vapor pressures (the equilibrium pressures exerted by volatile compounds) of these chemicals are summarized in Table 1.

**Table 1:** Chemical properties of chemicals sampled and separated by the monoGSA system^48^

Chemical	Kovats RI	Vapor Pr. At 25 °C [Pa]
tert-Butyl mercaptan	584	24130
Hexene	588	24490
Benzene	648	12640
PGME	673	1670
Toluene	755	3790
Tetrahydrothiophene	805	2450
Chlorobenzene	831	1600
PGMEA	857	520
Heptanal	883	470
1,3-dichlorobenzene	989	280

The ability of the system to monitor the change in mixture composition was characterized using Mixture 1 (hexene and heptanal) and Mixture 2 (PGME and PGMEA). During a chemical reaction, the percentage of the reagent decreases, whereas the percentage of the product increases. In this work, such a change in concentration was emulated using sample vials containing different concentrations of the reagent and product at 20 °C. Five analysis runs were performed at each concentration to assess the repeatability of the system (Fig. 3b–i).

As shown in the experimental results (Fig. 3d, h), the changes in peak height in the chromatograms tracked with the changes in the percentage of the reagent (hexene or PGME) and the product (heptanal or PGMEA) in the mixtures. The maximum variation in retention time for repeated tests performed with neat chemicals was ± 7.6% for hexene, ± 6.25% for heptanal, ± 6.10% for PGME, and ± 6.32% for PGMEA. The maximum relative standard deviation (RSD) for retention time was 4.7% across the four compounds. The variation in retention time was less than 13.5% for heptanal, PGME, and PGMEA across all concentrations tested. For hexene, the variation in retention time at a given concentration was below 6%, but there was a shift in retention time observed for mixtures with less than 50% hexene compared to neat hexene. The shift in retention time was caused by the column being overloaded with analytes, which changed the peak shape and retention time of the hexene peak^46^. The peak heights in response to each concentration were repeatable across the tests. The peak height for tests with neat chemicals was 79.2 ± 2.8 fF for hexene, 29.0 ± 1.3 fF for heptanal, 35.6 ± 1.0 fF for PGME, and 31.3 ± 0.4 fF for PGMEA; these results represented a variation of less than 2.7% (RSD) for the four chemicals. Comparable variation in peak height was observed across other concentrations. Hence, the monoGSA system has similar levels of repeatability in retention time and peak height as other μGC systems; a detailed comparison can be found in the Supporting Information Section S4.

The repeatability in retention time and peak heights indicates that the chromatograms can be used with a calibration curve to estimate the concentration of a chemical in the mixture. The calibration curve plots the ratio of normalized peak heights against the concentration of a chemical in the liquid mixture (Fig. 3e, i). The peak height has previously been shown to be highly proportional to the concentration of the chemical for capacitive detectors^14,15^ and this trend continues for the monoGSA system. The normalized peak height is defined as:

1 $$\hat{p}=\frac{p}{{p}_{Neat}}$$

where *p̂* is the normalized peak height, *p* is the peak height at the sampled concentration, and *p*_*Neat*_ is the peak height for the neat chemical. The peak height is normalized to linearize the relationship between the peak height and the concentration of the chemical and to reduce the uncertainty of concentration estimates across the full scale. A ratio of the peak heights is used because the concentration in the headspace sampled by the system is dependent on the vapor pressures of all chemicals in the mixture and is calculated using the equation:

2 $${P}_{1}=\frac{{\hat{p}}_{1}}{{\hat{p}}_{1}+{\hat{p}}_{2}}$$

where *P*_*1*_ is the ratio of normalized peak heights, *p̂*_*1*_ and *p̂*_*2*_ are the normalized peak heights for the chemicals in the mixture. The confidence interval (*CI*), which provides the estimated range of the average concentration value, and the prediction interval (*PI*), which provides the estimated range for concentration, are calculated using^47^:

3 $$CI={\hat{y}}_{h}\pm {t}_{(1-\alpha /2,n-2)}s\sqrt{\frac{1}{n}+\frac{{({x}_{0}-\bar{x})}^{2}}{\sum {({x}_{i}-\bar{x})}^{2}}}$$

4 $$PI={\hat{y}}_{h}\pm {t}_{(1-\alpha /2,n-2)}s\sqrt{1+\frac{1}{n}+\frac{{({x}_{0}-\bar{x})}^{2}}{\sum {({x}_{i}-\bar{x})}^{2}}}$$

where *ŷ*_*h*_ is the estimated mean, *t*_*(1-α/2,n-2)*_ is the t-distribution value at a specified confidence level and degree of freedom, *s* is the square root of the variance of the error term, *n* is the number of samples, *x*_*0*_ is the location of the prediction, *x̄* is the sample mean of *x*, and Σ*(x*_*i*_*- x̄)*^*2*^ is the sum of the squares of the difference between each *x* and the mean *x* value. The calibration curves can be used to estimate the percentage of a chemical or the peak height ratio. Using the prediction intervals, the concentration of the hexene and heptanal mixture can be calculated within a range of ±6.5% (Fig. 3e) and the concentration of PGME and PGMEA can be calculated within a range of ±8.5% (Fig. 3i).

#### Humidity response and range of analysis

**Fig. 4:**
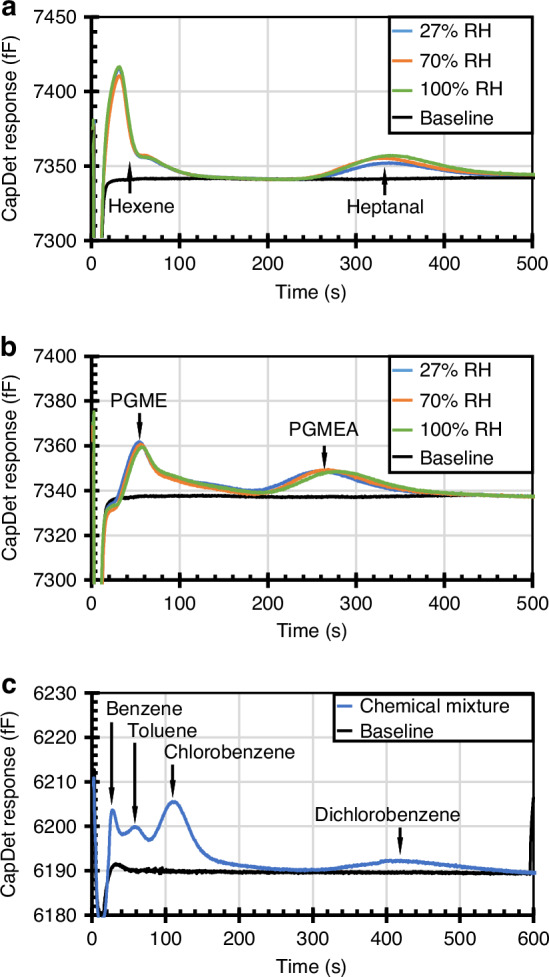
Experimentally obtained chromatograms. **a** Mixture 1 with varying humidity levels. **b** Mixture 2 with varying humidity levels. **c** Mixture 3

The impact of humidity on the monoGSA system was evaluated using three different relative humidity levels for both Mixture 1 and Mixture 2. The relative humidity in the ambient air was 15–30% at 20 °C, measured using a commercial humidity and temperature sensor (Part # 00215CA, AcuRite, WI, USA). A higher relative humidity (65-70%) was obtained by placing the sample vial and capillary tubes connected to the monoGSA chip ports into an enclosed container with a small amount of deionized water, allowing the humidity inside the container to evaporate fully, and measuring the humidity with the same commercial humidity sensor. A small amount of water was injected into the sample vial to achieve 100% relative humidity in the sampled chemical mixture. In the experimental results (Fig. 4a, b), the capacitive detector responses of the tested chemicals remained unchanged for all three relative humidity levels. Additionally, water peaks were not evident.

The ability of the monoGSA to separate chemicals with a broader volatility range was tested using Mixture 3 (with benzene, toluene, chlorobenzene, and 1,3-dichlorobenzene mixed in a liquid volume ratio of 1:1:1:7 and heated to 50 °C). The four chemicals in Mixture 3 were observed in the chromatogram (Fig. 4c). Results of neat chemical tests corroborated the identity of each peak, and the retention times of each chemical were at their expected locations based on the chemical’s Kovats retention index. This result preliminarily showed the monoGSA capability of analyzing chemicals with retention indices from as low as ≈650 (benzene) to as high as ≈1000 (1,3-dichlorobenzene).

### Passive and low-power sampling

**Fig. 5:**
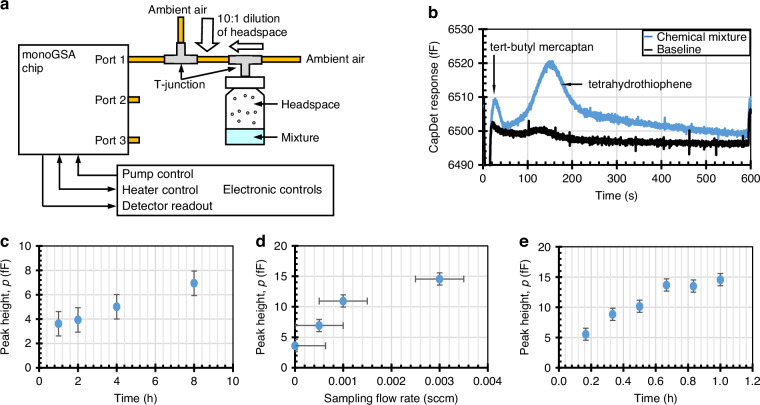
**Passive and low-power sampling results of the monoGSA. a** The test setup for passive and low-power sampling. **b** Chromatogram after passive sampling the diluted headspace of a mixture of tert-butyl mercaptan and tetrahydrothiophene for 8 h. Peak height of tetrahydrothiophene with (**c**) varied durations of passive sampling, (**d**) sampling for 1 h at different sampling flow rates, and (**e**) sampling with sampling pump operated at 20% power (corresponding to 0.003 sccm flow rate)

The passive and low-power sampling capabilities of the monoGSA were demonstrated using Mixture 4, composed of tert-butyl mercaptan and tetrahydrothiophene with a liquid volume ratio of 1:3. The headspace of the mixture was diluted by a factor of 10 using a T-junction (Fig. 5a) and then passively sampled by the monoGSA (Fig. 5b). The tert-butyl mercaptan, with its higher headspace concentration, saturated the preconcentrator after one hour of passive sampling. In contrast, the tetrahydrothiophene peak height varied linearly with the sampling time (Fig. 5c).

Low-power sampling was also tested with the sampling pump operated at 5%, 10%, and 20% of the standard KP1 operating power, corresponding to flow rates of 0.0005 sccm, 0.001 sccm, and 0.003 sccm, respectively (Fig. 5d). When sampling for one hour with low-power levels, the tetrahydrothiophene peak height varied linearly with sampling flow rates up to 0.001 sccm before starting to saturate at 15 fF. At 20% power, a similar linear relationship between peak height and sampling time was observed up to a sampling time of 40 min before saturation occurred (Fig. 5e). The response of tetrahydrothiophene under passive and low-powered sampling conditions reveals the promise of the monoGSA for applications where chemicals need to be sampled over a long period in an energy efficient manner.

## Conclusion

This effort describes an advancement and assessment of the first completely monolithic μGC system (the monoGSA) that integrates all the necessary analytical and fluidic components on the same chip, thereby achieving a self-sufficient gas phase analyzer that does not require any external fluidic components. In this system, three Knudsen pumps, a preconcentrator, a separation column, and a detector are monolithically integrated into a 15 × 15 mm^2^ μGC chip. This work reveals several important findings.

First, this work demonstrates that it is feasible to monolithically integrate Knudsen pumps into a μGC system and that design compromises must be considered. Suspended dielectric Knudsen pumps use thin, stress-balanced membranes to support a large number of pumping channels and provide the high thermal isolation needed for efficient gas flow, but are subject to mechanical fragility, narrow process windows, and complex fabrication^33–35^. In contrast, this work uses the robust Knudsen pump designs with pumping channels formed in unsuspended silicon and oxide layers, sacrificing thermal isolation and the number of pumping channels for significantly improved yield, fabrication simplicity, and integration compatibility^36^. This design shift in Knudsen pump design aims to relax the fabrication complexity for the pumps to better accommodate the added challenges of monolithically integrating other fluidic components in the μGC system. Pumping channels of the Knudsen pump are positioned to allow for sufficient heat sinking to establish the temperature gradient needed for Knudsen pump operation.

Second, this work demonstrates that even a small flow rate is enough to support µGC analysis, which can meet the needs of certain applications. Operating at 0.5 W/channel, a temperature gradient of 45 °C was achieved across the pumping channel of the Knudsen pump with thermal isolation from the oxide layer and heat sinking from the bulk silicon^36^. When integrated in the monoGSA chip, this temperature gradient enabled the Knudsen pump to provide a flow rate of 0.009 sccm through the separation flow path. Chemical separation at this flow rate was achieved by using a separation column with a smaller channel height of 40 μm, as opposed to a height of 250 μm in our earlier μGC system^15^, to reduce the hydraulic diameter and increase the flow velocity to an estimated value of 1.88 cm/s.

Third, flow direction control can be achieved by multiple pumps without using valves. This operation had been previously reported^14^ and is expanded in this work, where a unique arrangement of the three Knudsen pumps is used to provide counter-balanced flow rates and therefore achieve valveless flow control for sampling and separation. Our earlier μGC system also operated without valves but relied on a bi-directional Knudsen pump module to route both sampling and separation flows through the same path^15^. The architecture in this work introduces a dedicated sampling path that bypasses the separation components. Bypassing these components not only reduces the flow resistance, thus enabling higher sampling flow rates, but also eliminates the possibility of undesired analyte retention in the separation column and detector during sampling. Additionally, this system uses unidirectional Knudsen pumps instead of bi-directional ones, simplifying fabrication and operation while maintaining the valveless nature of the system by generating counterbalancing pressure heads that block undesired gas flow in unused flow paths. KP1 and KP3 provide the sampling and separation flows, respectively, and the blocking pressures of KP3 and KP2 are sufficient to provide counterbalancing pressure heads that prevent additional gas from entering the system during operation.

Fourth, even with the thermal conductive silicon substrate, thermal crosstalk between μGC components can be effectively reduced by careful thermal isolation and management. Thermal isolation is achieved by introducing cutouts in the substrate to reduce thermal conduction between components and is further enhanced by shunting heat to the silicon substrate and thermally grounding the substrate with the heat sink and heat pipe. These strategies prove effective, enabling a temperature difference of over 60 °C between the preconcentrator and the other μGC components during desorption: while the preconcentrator reaches 130 °C, the column and other components remain below 60 °C and cool to under 40 °C within 5 s, preserving the separation column and detector performance.

Overall, the monoGSA marks a major step forward in μGC miniaturization, moving beyond hybrid and semi-integrated systems toward a fully integrated, compact solution. System-level vapor sampling and chromatographic analysis are demonstrated, showing promise for applications that involve a small number of known chemical species and require quantification of these species, e.g., chemical reaction process monitoring. The monoGSA was experimentally evaluated with representative reagents and products from hydroformylation, esterification, and chlorination reactions. The device demonstrated high repeatability (*viz*., relative standard deviation of <2.7% in peak height, <4.7% in retention time), allowed for the concentration estimation to within a range of ±6.5–8.5%, and stable operation across relative humidity levels from 15% to 100% with no visible water interference. The system resolved analytes spanning Kovats retention indices from ≈650 to ≈1000 using the 4.5 cm long on-chip column under low-flow conditions. Passive and low-power sampling operation of the monoGSA was also validated, highlighting the system’s suitability for energy-efficient, long-duration monitoring. These results indicate that monoGSA may be well suited for continuous, real-time process monitoring.

The monoGSA platform offers significant potential for further optimization to expand its chemical detection capabilities. Future efforts will focus on improving Knudsen pump performance and exploring alternative preconcentrator and column materials. In order to evaluate pathways for performance improvement, the separation performance of the microfabricated column was individually evaluated, as described in Section S5 of the Supporting Information. Based on the Golay plot, the optimal flow rate of the column was higher than the actual flow rate of 0.009 sccm provided by KP3 for separation, indicating potential for further improvement in performance. Enhancing the pump design by adding more parallel pumping channels can increase the total flow rate while reducing overall flow resistance across the pump structure. Since the separation flow rate is jointly determined by pump performance and flow resistance, such improvements can enable higher flow rates, thereby enhancing separation efficiency. Additional pumping channels introduce greater thermal dissipation challenges, which can be easily addressed by increasing the pump’s footprint, incorporating more effective passive cooling strategies, or employing active solutions such as Peltier elements. Improvements to the pumping channels of the Knudsen pumps should ultimately increase the temperature difference between the hot and cold ends of the channel by adjustments to the layout and process sequence, such as by moving the heaters closer to the channel edges, using a thin film thermally insulating layer along the channel (as previously reported by our team^33^), and strategically placing thermal isolation trenches to further reduce parasitic heat loss while maintaining mechanical robustness.

In parallel with flow and Knudsen pump optimization, tuning the system for analytes of varying volatility will broaden the chemical coverage. For lower vapor pressure compounds, increasing the retention characteristics of the preconcentrator, such as through tailored sorbent materials, can improve analyte capture during sampling and increase detection sensitivity. Conversely, for higher vapor pressure species, improving separation can be achieved by modifying the stationary phase chemistry or extending the column length to allow better resolution. Improvements to thermal isolation, such as expanding the thermal isolation region or transitioning the preconcentrator to a suspended structure, can also enhance separation efficiency, particularly at the start of separation, as this further reduces the impact of preconcentrator desorption on the column and detector. These refinements chart a clear path toward extending the monoGSA system’s performance and utility across a broader range of applications.

## Supplementary information

Supporting Information
