# Supplementary material for: Monolithic integration of Knudsen pumps to form a complete, self-sufficient fluidic system for microscale gas chromatography
Source: Microsyst Nanoeng. 2025 Dec 9;11:242. doi: 10.1038/s41378-025-01091-2 (PMC12686500; doi:10.1038/s41378-025-01091-2)
Supplement: Supplementary file 1 — Supporting Information [file 41378_2025_1091_MOESM1_ESM.pdf]

Supporting Information

Monolithic Integration of Knudsen Pumps to Form a Complete, Self-Sufficient Fluidic System for Microscale Gas Chromatography

Xiangyu Zhao[1,3], Tsenguun Byambadorj[1,3], Tao Qian[1,3], Qu Xu[2,3], Declan Winship[1,3], Yingkun Ma[1,3], Yutao Qin [1,3,*], Yogesh B. Gianchandani[1,3,*]

[1]Department of Electrical Engineering and Computer Science, University of Michigan, Ann Arbor, MI 48109, USA

[2]Department of Integrative Systems + Design, University of Michigan, Ann Arbor, MI 48109, USA

[3]Center for Wireless Integrated MicroSensing and Systems (WIMS^2), University of Michigan, Ann Arbor, MI 48109, USA

*Corresponding authors

Email: Y.Q. (yutaoqin@umich.edu); Y.B.G. (yogesh@umich.edu)

Contents

S1. Flow Resistance and Knudsen Pump Performance
S2. Thermal Simulation of the monoGSA System
S3. Circuit Schematics of monoGSA System
S4. Retention Time and Peak Height Repeatability
S5. Evaluation of the Separation Column

S1. Flow Resistance and Knudsen Pump Performance

To determine the flow resistance of the components in the monoGSA, an external pump (#MP6-gas, Servoflo, MA, USA) was used to provide a flow through one of the three flow paths in the system while blocking the remaining port. For example, when a flow from Port 1 to Port 3 was provided to measure the flow resistance of the separation path, Port 2 was blocked. The ports for the monoGSA system were labeled based on the Knudsen pump closest to each port (e.g., the port at KP1 would be labeled as Port 1). The flow rate was measured using a flow meter (MW-5SCCM-D/5M, Alicat Scientific, Inc., AZ, USA), while the pressure drop across the fluidic path was measured with a differential pressure sensor (MPX5010DP, Freescale Semiconductor Inc., TX, USA). The measurement uncertainties were estimated to be ±0.001 sccm for the flow rate and ±1 Pa for the pressure. This test was repeated for all three monoGSA flow paths.

The total flow resistance along each path was calculated as the ratio of the pressure drop to the flow rate. The sampling flow path (from Port 2 to Port 1), the separation path (from Port 1 to Port 3), and the path from Port 2 to Port 3 showed flow resistances of 37.0 kPa/sccm, 49.5 kPa/sccm, and 55.2 kPa/sccm, respectively. The measured flow resistance for a path was set equal to the sum of flow resistances of the components in the path (*i.e.* the separation path flow resistance is equal to the sum of the flow resistance of KP1, the preconcentrator, the column and detector, and KP3) and the system of equations formed by the equation for the three separate used to calculate the flow resistance of each component (Table S2). All pumping channels in the three

Knudsen pumps were assumed to have equal flow resistances. Based on the results of this analysis, the flow resistance of the preconcentrator was 1.4 kPa/sccm, the flow resistance of the separation column and detector combined was 19.6 kPa/sccm, and the flow resistances of KP1, KP2, and KP3 were 14.3 kPa/sccm, 21.4 kPa/sccm, and 14.3 kPa/sccm, respectively. The Knudsen pumps in each flow path contributed significantly to the flow resistance.

Table S1: Measured flow resistance of monoGSA components

**Component**	**Flow Resistance (kPa/sccm)**
Preconcentrator	1.4
Column and Detector	19.6
KP1	14.3
KP2	21.4
KP3	14.3
Sampling flow path (Port 2 to Port 1)	37.0
Separation flow path (Port 1 to Port 3)	49.5
Port 2 to Port 3	55.24

The pumping characteristics of KP1 and KP3 were estimated from experimental testing of a separate, standalone six-channel Knudsen pump with the same pump design and co-fabricated with the monoGSA design on the same SOI wafer. The standalone six-channel Knudsen pump provided a maximum flow rate of 0.037 sccm and a blocking pressure of 340 Pa when powered at 2.10 W (0.35 W per pumping channel). Increasing the power applied to the pumps improved the

flow rate and blocking pressure of the pump. The upper limit in power was set to ≈0.5 W per pumping channel to protect the heater traces from damage. Based on these results and the measured flow resistances, the output flow and pressure head characteristics of the Knudsen pump and the flow resistances of the sampling and separation paths were plotted (Fig. S1). The intercept between the Knudsen pump performance line at 2.10 W and the two flow resistance lines indicated the expected flow rates to be 0.011 sccm for sampling and 0.008 sccm for separation.

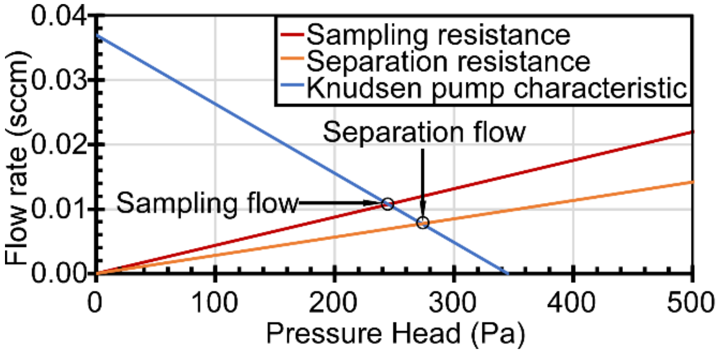

Fig. S1: Flow resistance of the sampling and separation flow paths and the estimated Knudsen pump performance. The expected flow rates are 0.011 sccm for sampling and 0.008 sccm for separation.

Experimentally measured flow rates of the monoGSA system matched the expectation. When KP1 was powered by 2.10 W to provide the sampling flow and KP3 was powered by 0.84 W to resist a flow through Port 3, the sampling flow rate was measured at 0.011 sccm and the flow through Port 3 was measured at 0 sccm. When KP3 was powered by 2.10 W to provide the separation flow and KP2 was powered by 0.61 W to resist a flow through Port 2, the separation flow rate was measured at 0.009 sccm and the flow through Port 2 was minimized. The power

required for the pump to resist unwanted air flow is dependent on the flow rate in the main path and was obtained by adjusting the power applied to the pump that resists flow until a flow rate is correct.

S2. Thermal Simulation of the monoGSA System

The monoGSA temperature distribution during preconcentrator desorption was analyzed by electrically and thermally coupled solid mechanics models in COMSOL Multiphysics®. The thermal simulation includes the monoGSA chip stack containing a SOI and two fused silica chips with a heat sink on the top of the chip and a heat pipe on the bottom connected to the chip using a layer of thermal interface material. The critical parameter values used in the model are listed in Table S2. The heat sink fins and the far end of the heat pipe are set at 35°C and 30°C, respectively, which were the experimentally measured values when operating the Knudsen pump at 0.4 W/channel. Thermal isolation cutouts in the handle silicon surrounding the preconcentrator area is added to reduce the thermal conductivity between the preconcentrator and the rest of the chip. Preconcentrator heater metal traces are drawn with the dimensions consistent with the actual preconcentrator and joule heating with a 26.5 V voltage pulse was applied to the heater during 2-20 s to simulate the preconcentrator desorption.

Table S2: Material properties and thicknesses assumed in the FEA simulation

**Structure**	**Material**	**Thickness (μm)**	**Thermal Conductivity (W/mK)**	**Resistivity (Ω·m)**	**TCR (K^{-1})**
Upper oxide	SiO_2	2	1.4	-	-
Device silicon	Si	12	130	-	-
Buried oxide	SiO_2	0.38	1.4	-	-
Handle layer	Si	525	130	-	-
Metal 1	Ti/Pt	0.03/0.1	76	4.0×10^{-7}	1.3×10^{-3}
Fused silica dies	Fused silica	700	1.4	-	-
Thermal interface pad	Silicone polymer	1000	12.5	-	-
Heat sink	Al	2500	237	-	-
Heat pipe	Cu	2500	401	-	-

The model incorporated joule heating at the preconcentrator heater and the resulting temperatures of the preconcentrator and the column were averaged over their designed areas. As shown in the simulation results (Fig. S2), the preconcentrator was heated rapidly from 25°C to 105°C during 2-4 s and further to 114°C during 4-20 s, a thermal response time of 2 seconds which is sufficient for desorption. The column temperature remained below 60°C during the desorption and fell quickly below 40°C within 5 seconds afterwards. The other μGC components underwent similar temperature profiles. As evident from the simulation results, the thermal dissipation from the heat sink and heat pipe allowed sufficiently high temperatures for thermal desorption while maintaining low levels of thermal crosstalk to the rest of the chip. Experimental validation of the temperature simulation was performed by heating the preconcentrator at 26.5 V for 18 seconds and measuring the temperature with the thermistor fabricated on the preconcentrator (Fig. S3). The measured temperatures closely matched the simulation results throughout the heating period.

However, the experimental results reached a final temperature of 127°C, which was 13°C (10.2%) higher than the simulation results. This discrepancy can be attributed to the practical non-ideality in the heat dissipation that was not represented in the simulation.

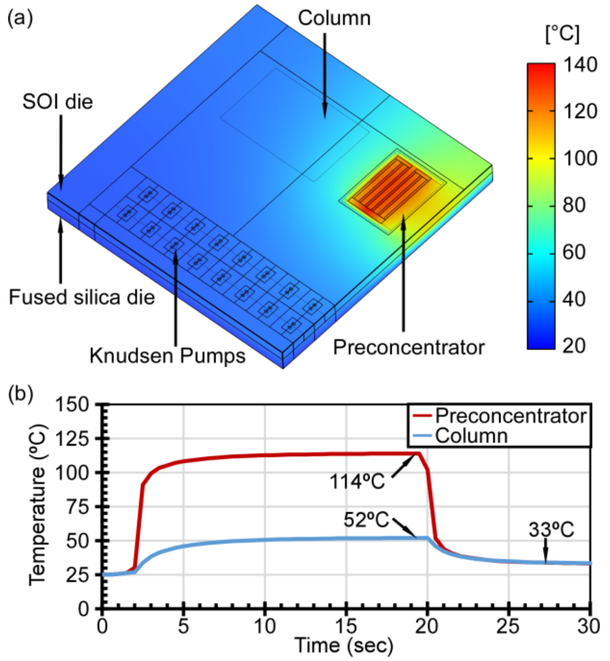

Fig. S2: The simulated temperature distribution during preconcentrator desorption. (a) Simulated temperature distribution at the end of preconcentrator desorption. (b) Averaged preconcentrator and column temperatures during desorption.

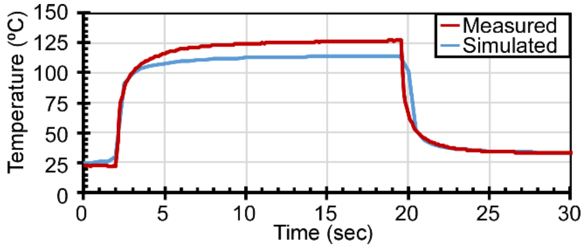

Fig. S3: Experimental validation of the preconcentrator temperature during desorption.

S3. Circuit Schematics of monoGSA System

The monoGSA system uses a Raspberry Pi (RPi) to perform system control and data readout. The heating of the preconcentrator, column, and three Knudsen pumps are controlled

using an analog control circuit implemented using a digital potentiometer (# MCP45HVX1, Microchip Technology, Chandler, AZ, USA) that controls the output voltage of a buck converter (# TPS54061DRBR, Texas Instruments, Dallas, TX, USA) (Fig. S4a). The capacitance readout is performed using a high-resolution capacitance-to-digital converter (CDC) (#AD7746ARUZ, Analog Devices Inc., Wilmington, MA, USA) (Fig. S4b). The selected CDC chip provides high tolerance to parasitic capacitance between the sensing electrode and ground which removes impact of parasitic capacitance on the detector reading. The capacitive-to-digital converter, when properly configured and operated in differential sensing mode, has been confirmed in our separate internal tests to achieve an RMS noise as low as 0.013 fF. Temperature measurements are made by reading the resistance of on-chip thermistors located at the preconcentrator, column, Knudsen pumps, and two bulk silicon locations using a voltage divider coupled to an ADC (#ADS1115, Texas Instruments, Dallas, TX, USA) (Fig. S4c). Closed-loop control of heating and pumping could be realized using these temperature measurements. To power the system, 24 V from a wall supply along with several DC-DC converters are used (Fig. S4d). The 24 V is directly used to generate the analog voltages required for heating different μGC components and also used to provide a $V_{Digital}$ of 5 V through a DC-DC converter (PDQE20-Q24, CUI Inc., Tualatin, OR, USA). The $V_{Digital}$ (5 V) is used to provide a 3.3 V supply, i.e., $V_{Digital}$ (3.3 V), using a voltage regulator (NCP51460, Onsemi, Scottsdale, AZ, USA) and is also passed through a Pi filter to provide a low-noise 5 V supply, i.e., V_{Analog} (5 V). The V_{Analog} (5 V) is used to provide a low-noise 3.3 V supply,

i.e., V_{Analog} (3.3 V), and a 1.2 V reference voltage for the ADC using a voltage reference chip (LM4041, Texas Instruments, Dallas, TX, USA).

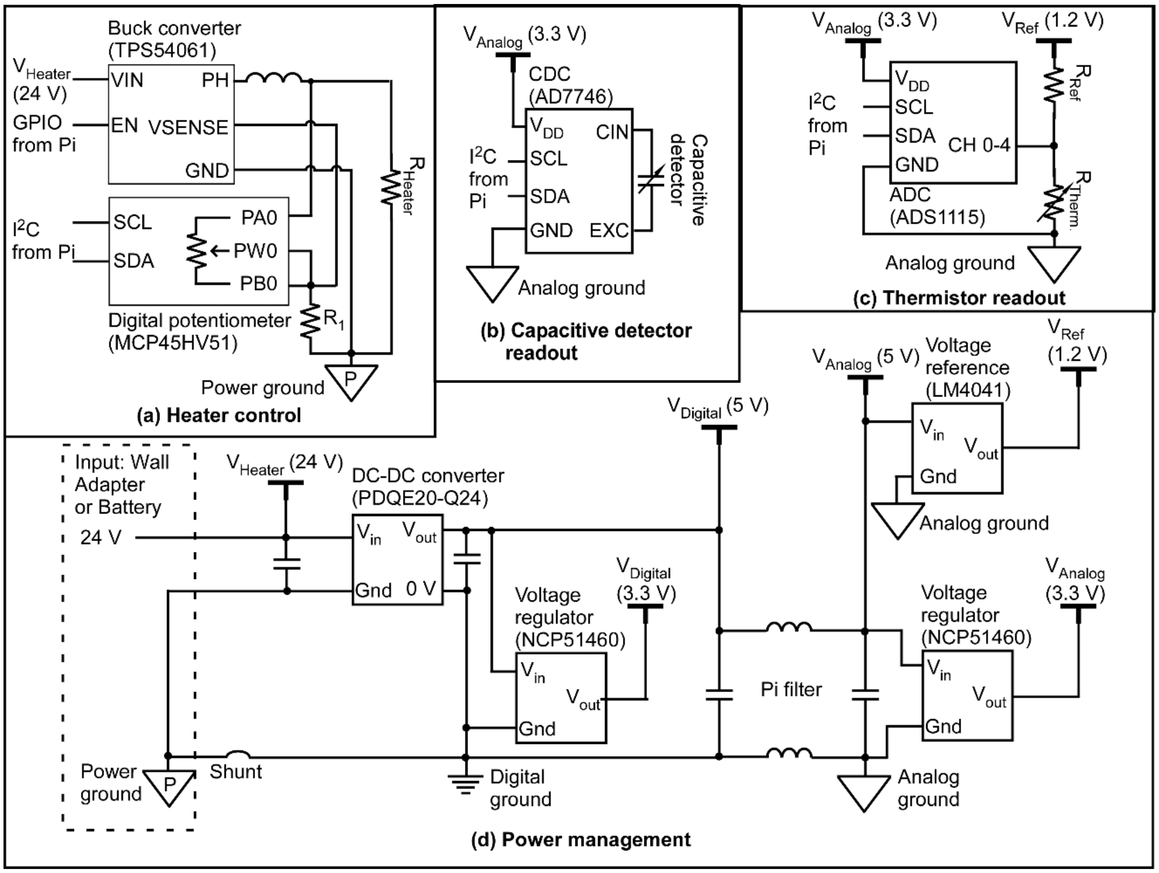

Fig. S4: The circuit schematics of the monoGSA system: a) Heater control to generate a stable, low ripple voltage. b) Capacitive readout circuit implemented using a high-resolution capacitance to digital converter. c) Thermistor readout using a voltage bridge and ADC for temperature measurement. d) Power management to generate the voltages needed for heating and to power microsystem electronics.

S4. Retention Time and Peak Height Repeatability

For the monoGSA, the relative standard deviation (RSD) for retention time were 4.6% for hexene, 2.2% for heptanal, 4.7% for PGME, and 1.4% for PGMEA. The RSD values were 2.4%

for hexene, 2.7% for heptanal, 2.0% for PGME, 1.1% for PGMEA. A comparison of the retention time and peak height repeatability is shown in Table S3. Based on the reported repeatability results, our work demonstrates retention time RSDs (1.4–4.7%) and peak height RSDs (1.1–2.7%) that are comparable to previously reported systems.

Table S3. Benchmark of retention time and peak height repeatability against state of the art μGC systems.

	Retention Time (RSD %)	Peak height (RSD %)
Collin et al. [S1]	3.6%	11.0%
Wang et al. [S2]	0.3-5.9 %	0.6-5.6
You et al. [S3]	0.1%	4.5%
Winship et al. [S4]	1.4-1.7%	-
This work	1.4-4.7 %	1-2.7 %

S5. Evaluation of the Separation Column

To further investigate the separation condition employed in the system operation, the separation performance of the microfabricated column was individually evaluated with a benchtop GC (#Agilent 7890, Agilent, CA, USA). For this test, Port 1 was blocked with a septum, KP2 was connected to the benchtop GC inlet, and KP3 was connected to the flame ionization detector (FID). A mixture of propylene glycol methyl ether (PGME) and propylene glycol monomethyl ether acetate (PGMEA) was injected into the benchtop GC inlet and carried by 0.020 sccm N_2 flow through the monoGSA chip, which was maintained at 40ºC inside the benchtop GC oven.

The separation performance of the column can be represented by the number of theoretical plates (*N*) and the height equivalent to a theoretical plate (*HETP*), which can be calculated from the chromatogram of an isothermal separation experiment [S5]:

$$N = 5.54\left(\frac{t_R - t_0}{PWHH}\right)^2 \quad (1)$$

$$HETP = \frac{L}{N} \quad (2)$$

where t_R is the retention time of the chemical, t_0 is the retention time of an unretained analyte, *PWHH* is the peak width at half height, and L is the length of the column. The t_R and *PWHH* were obtained from the chromatogram, while t_0 was estimated from the flow rate and dimensions of the separation and guard columns. A higher column efficiency is indicated by a lower HETP, which is affected by the average flow velocity of the carrier gas.

To characterize the *HETP* dependence on the flow velocity, the aforementioned test of the separation column was repeated with the carrier gas flow rate swept from 0.01 sccm to 0.4 sccm. As shown from the results, the optimal flow rate for PGME was 0.090-0.110 sccm, at which the *HETP* was 0.33 mm as calculated from a measured t_R of 50.3 s, a measured *PWHH* of 1.0 s, and a calculated t_0 of 29.1 s (Fig. S5a). The optimal flow rate for PGMEA was also 0.090-0.110 sccm, at which the HETP was 0.16 mm as calculated from a measured t_R of 72.3s, measured *PWHH* of 6.1 s, and a calculated t_0 of 29.1 s (Fig. S5b). Note that the optimal flow rate of the column was higher than the actual flow rate of 0.009 sccm used by the monoGSA for separation, indicating that further performance improvement can be achieved by increasing the separation flow rate.

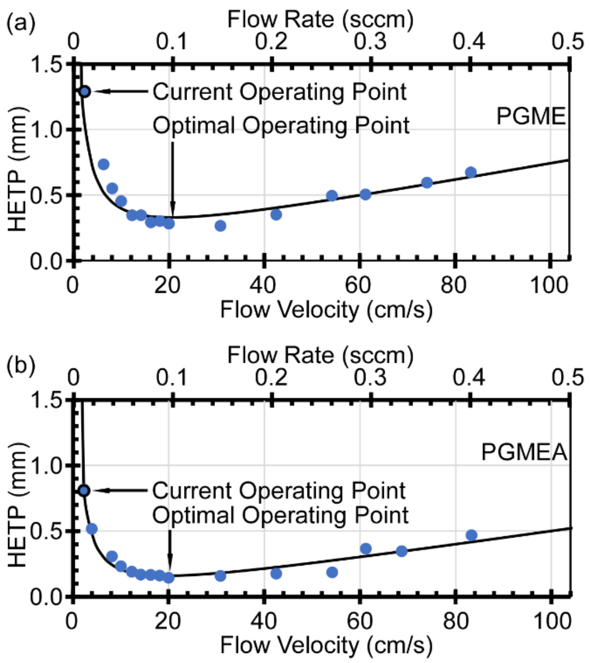

Fig. S5: Golay plot of the separation column calculated from injection tests of (a) PGME and (b) PGMEA. The optimal flow rate is higher than the operational flow rate of the monoGSA system, indicating potential for further performance improvement.

References

[S1] Collin, W. R. *et al.* Microfabricated Gas Chromatograph for Rapid, Trace-Level Determinations of Gas-Phase Explosive Marker Compounds. *Analytical Chemistry* **86**, 655-663 (2014).

[S2] Wang, J. *et al.* Belt-Mounted Micro-Gas-Chromatograph Prototype for Determining Personal Exposures to Volatile-Organic-Compound Mixture Components. *Analytical Chemistry* **91**, 4747-4754 (2019).

[S3] You, D.-W. *et al.* A portable gas chromatograph for real-time monitoring of aromatic volatile organic compounds in air samples. *Journal of Chromatography A* **1625**, 461267 (2020).

[S4] Winship, D. *et al.* An enhanced-performance multisensing progressive cellular µGC: design advances and blind test results. *Microsystems & Nanoengineering* **11**, 141 (2025).

[S5] Grob, R. L. & Barry, E. F. *Modern practice of gas chromatography*. (John Wiley & Sons, 2004).
